# More than meat: contributions of livestock systems beyond meat production

**DOI:** 10.1093/af/vfab006

**Published:** 2021-05-17

**Authors:** Andrea J Garmyn

**Affiliations:** Departments of Food Science and Human Nutrition, Animal Science, Michigan State University, East Lansing, MI 48824, USA

Global meat production topped over 340 million tons in 2018, representing over 80 billion animals (Ritchie and Poser, 2019). An estimated 69 billion chickens, 656 million turkeys, 1.5 billion pigs, 302 million cattle, 574 million sheep, and 479 million goats were slaughtered worldwide for meat production in 2018 (Ritchie and Poser, 2019). Yet, these numbers represent only a small fraction of the total population, especially of the larger red meat species, presented as a snapshot of livestock counts at a given time in a year (2018) in Table 1 (FAO, 2020). A large portion of the global livestock population is not intended for meat production, at least not as its primary purpose. Moreover, depending on the species, 35% to 50% of the live weight of slaughtered animals is not used for human food (Meeker and Hamilton, 2006).

**Table 1.** Global livestock counts for selected species at a given time in 2018

Species	Number of animals
Buffaloes	233,719,311
Camels	35,848,571
Cattle	1,553,162,432
Chickens	29,079,694,000
Goats	1,184,298,887
Horses	61,219,008
Pigs	1,425,507,453
Sheep	1,373,546,174
Turkeys	466,872,000
Total	35,413,867,836

The goal of this issue is to the highlight the ways that the livestock industry and products from livestock production can be more than just meat. There are tangible products from the livestock industry, including milk, eggs, and edible by-products, and also nonfood items, such as fiber and a wide array of rendered products. There are also many intangible benefits from livestock production such as direct and indirect employment opportunities in developed countries or as wealth or assets in developing countries. Some livestock are even used to help maintain rangelands for fire control or “upcycle” food waste, converting otherwise inedible foodstuffs into high-quality protein. Lastly, livestock production can play a critical role in youth development through programs such as 4-H and FFA, where children and young adults gain valuable life skills through raising and showing livestock as 4-H/FFA projects, in addition to youth and collegiate livestock judging activities. We would like to communicate the integral role that livestock play in the overall food and environmental system.

When asked what products that are derived from livestock, I think many would immediately recognize meat, milk, and eggs are sourced from animals. But what about the products other than those intended for human consumption? Doyle et al. (2021) provide an overview of the wool industry, highlighting the importance and value of wool production from sheep. Where else would we turn to other than Australia to provide this insightful review that covers fiber production on a biological level, on-farm determinants of productivity and profitability, and wool characteristics and processing.

What happens to the 35% to 50% of the animal that is not used for human food (Figure 1; Meeker and Hamilton, 2006)? Wilkinson and Meeker (2021) explain the process of agricultural rendering and discuss the “Big 4” rendered product markets: pet food/animal feed, fuel, oleochemical products, and fertilizer. Wilkinson and Meeker (2021) also highlight how rendering supports the three pillars of sustainability: environmental, economic, and social.

**Figure 1.**
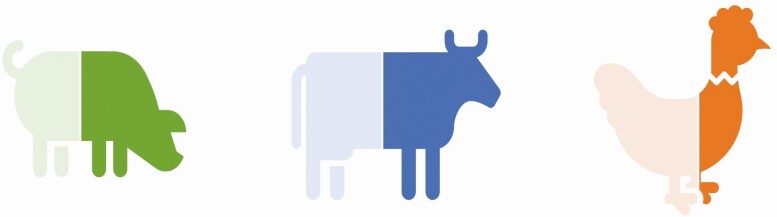
Thirty-five to 50% of the live weight of animals harvested in the United States is not used for human food. For example, 45% of the live weight of hogs, 49% of the live weight of cattle, and 37% of the live weight of chickens are not consumed by humans (Meeker and Hamilton, 2006).

Next, we’ll delve into those intangible benefits—the ones we cannot sink our teeth into or hold in our hands. The meat supply chain provides so much more than just nourishment to those consuming animal-derived products, specifically meat. Dr. Phil Bass (2021) highlights the employment and economic benefits tied directly and indirectly to the meat supply chain. Bass (2021) focuses on three main areas in this assessment—live animal transportation, packing house personnel and material, and the restaurant dining industry.

In a series of contributions from Africa led by Dr. Liveness Banda, she discusses how livestock provide so much more than simply the food they produce in developing countries. Banda and Tanganyika (2021) highlight the nonfood roles of livestock in developing countries, including working animals for draught power, pest and weed control, social status or prestige, and a form of savings. Next, she focuses specifically on smallholder dairy farmers, focusing on the ways dairy farming contributes more than milk for income to rural livelihoods (Banda et al., 2021). Dairy farmers often allocate land to food crops, cash crops, and pasture production, giving them more diversified sources of income and making them more resilient to food insecurity than nondairy farmers.

In a contribution from Spain, Insausti et al. (2021) describe European horse meat production systems. Equine farms play an essential socioeconomical role in the environmental preservation of mountain areas in many regions of southern Europe, since horses can efficiently digest cellulose. Although horse meat production (and consumption) is considered taboo in some areas around the world, these extensive systems play an important role in the sustainable development of mountain areas mainly through biomass management and subsequent fire prevention.

Ominski et al. (2021) offer a Canadian perspective to livestock upcycling. Livestock have the capability to utilize by-products and food waste and convert these low-value materials into high-quality protein. Although there is great potential, challenges do exist. Ominski et al. (2021) also discuss how to navigate regulatory restrictions, safety concerns, and logistical considerations for by-product or food waste utilization in livestock feeding systems.

Lastly, livestock production can play a critical role in youth development through programs like 4-H and FFA, where children and young adults gain valuable life skills through raising and showing livestock as 4-H/FFA projects, in addition to youth and collegiate livestock judging activities. When Dr. Anna Dilger and I were discussing the theme and articles we wanted to feature for this issue, the relationship between livestock production and youth development is one topic we were both intrigued and excited about. Even when I started initial discussions with Dr. Clint Rusk, I believe his words were “It’s an article that needs to be written.” Martin and Rusk (2021) go beyond these youth organizations and focus specifically on the benefits of 4-H livestock judging. Youth and young adults develop several life skills from their participation in judging activities—problem solving, decision making, dealing with pressure, self-motivation, self-discipline, organization, teamwork, and communication. It is a long list, and these skills are often sought after by future employers.

In closing, Dr. Anna Dilger and I would like to thank all of the authors around the globe for their contributions and also the reviewers who assisted in their publications. We hope this issue sheds some light on the nonfood roles of the livestock production and offers some regional perspective on those roles.

## About the Author

**Andrea Garmyn** is an academic specialist in Meat Science Teaching and Outreach at Michigan State University. Garmyn holds a split appointment between the Departments of Food Science and Human Nutrition and Animal Science, where her current role focuses on undergraduate meat science instruction. After 10 yr, Garmyn left Texas Tech University, where she served as a research assistant professor for several years. Andrea’s research involves beef and lamb eating quality and implementing strategies during animal production or postmortem processing to improve eating quality and ensure positive consumer outcomes.
